# P-471. Approaches to Invasive Fungal Diseases in Pediatric Cancer Centers: An Analysis of Current Practices and Challenges in Germany, Austria, and Switzerland

**DOI:** 10.1093/ofid/ofaf695.686

**Published:** 2026-01-11

**Authors:** Danila Seidel, Zoi-Dorothea Pana, Daniel Ebrahimi-Fakhari, Sarina K Butzer, Katrin Mehler, Ilana Reinhold, Arne Simon, Christian Dohna-Schwake, Ines Mack, Nicole Bodmer, Tim Niehues, Oliver A Cornely, Andreas H Groll, Thomas Lehrnbecher

**Affiliations:** University Hospital of Cologne, Cologne, Nordrhein-Westfalen, Germany; Medical School, University of Nicosia (UNIC), Nicosia, Cyprus, Nicosia District, Nicosia, Cyprus; Infectious Disease Research Program, Center for Bone Marrow Transplantation and Dept. of Pediatric Hematology/Oncology, University Children’s Hospital Münster, Germany, Muenster, Nordrhein-Westfalen, Germany; University of Cologne, Faculty of Medicine and University Hospital Cologne, Department of Pediatrics, Division of Pediatric Infectious Diseases, Cologne, Germany; University of Cologne, Faculty of Medicine and University Hospital Cologne, Department of Pediatrics, Division of Pediatric Hematology and Oncology, Cologne, Germany, Cologne, Nordrhein-Westfalen, Germany; University of Cologne, Faculty of Medicine and University Hospital Cologne, Department of Pediatrics, Division of Pediatric Infectious Diseases, Cologne, Germany; University of Cologne, Faculty of Medicine and University Hospital Cologne, Department of Pediatrics, Division of Pediatric Hematology and Oncology, Cologne, Germany, Cologne, Nordrhein-Westfalen, Germany; Faculty of Medicine and University Hospital Cologne, Institute of Translational Research, Cologne Excellence Cluster on Cellular Stress Responses in Aging-Associated Diseases (CECAD), University of Cologne, Cologne, Germany; Department I of Internal Medicine, Faculty of Medicine and University Hospital Cologne, Excellence Center for Medical Mycology (ECMM), University of Cologne, Cologne, Germany, Cologne, Nordrhein-Westfalen, Germany; University of Würzburg, Wurzburg, Baden-Wurttemberg, Germany; Department of Pediatrics I, Neonatology, Pediatric Intensive Care, Pediatric Infectiology, Pediatric Neurology, University Hospital Essen, University Duisburg-Essen, Essen, Germany, Essen, Nordrhein-Westfalen, Germany; Department of Paediatric Infectious Diseases and Vaccinology, University Children's Hospital Basel (UKBB) and University of Basel, Basel, Switzerland, Basel, Basel-Stadt, Switzerland; Department of Oncology, University Children's Hospital Zurich, Switzerland, Zurich, Zurich, Switzerland; Centre for Child and Adolescent Health, Helios Klinikum Krefeld, Germany, Krefeld, Nordrhein-Westfalen, Germany; University of Cologne, Faculty of Medicine and University Hospital Cologne, Cologne, Nordrhein-Westfalen, Germany; University Children’s Hospital Muenster, Muenster, Germany, Muenster, Nordrhein-Westfalen, Germany; Division of Pediatric Hematology and Oncology, Hospital for Children and Adolescents, University Hospital, Johann Wolfgang Goethe University, Frankfurt am Main, Germany, Frankfurt/Main, Nordrhein-Westfalen, Germany

## Abstract

**Background:**

Invasive fungal diseases (IFDs) cause high morbidity and mortality in children with cancer and hematopoietic cell transplantation (HCT). While international guidelines exist for prevention, diagnosis, and treatment, pediatric-specific evidence remains limited and practices vary.

Geographic distribution of the 62 participating pediatric oncology centers

**Figure:**
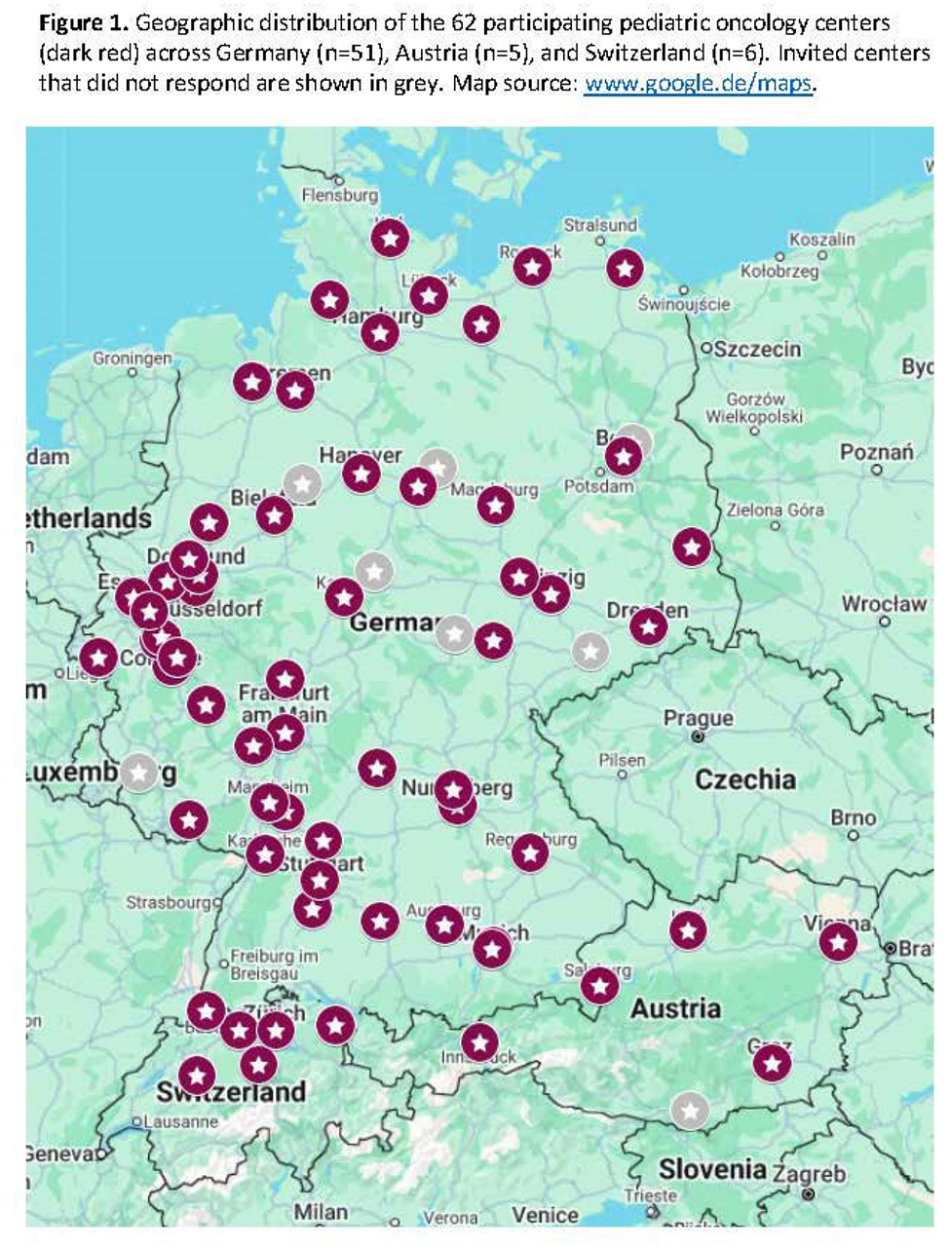

First-line and first-line alternative antifungal treatment for candidemia and invasive pulmonary aspergillosis in 62 pediatric oncology centers

**Figure:**
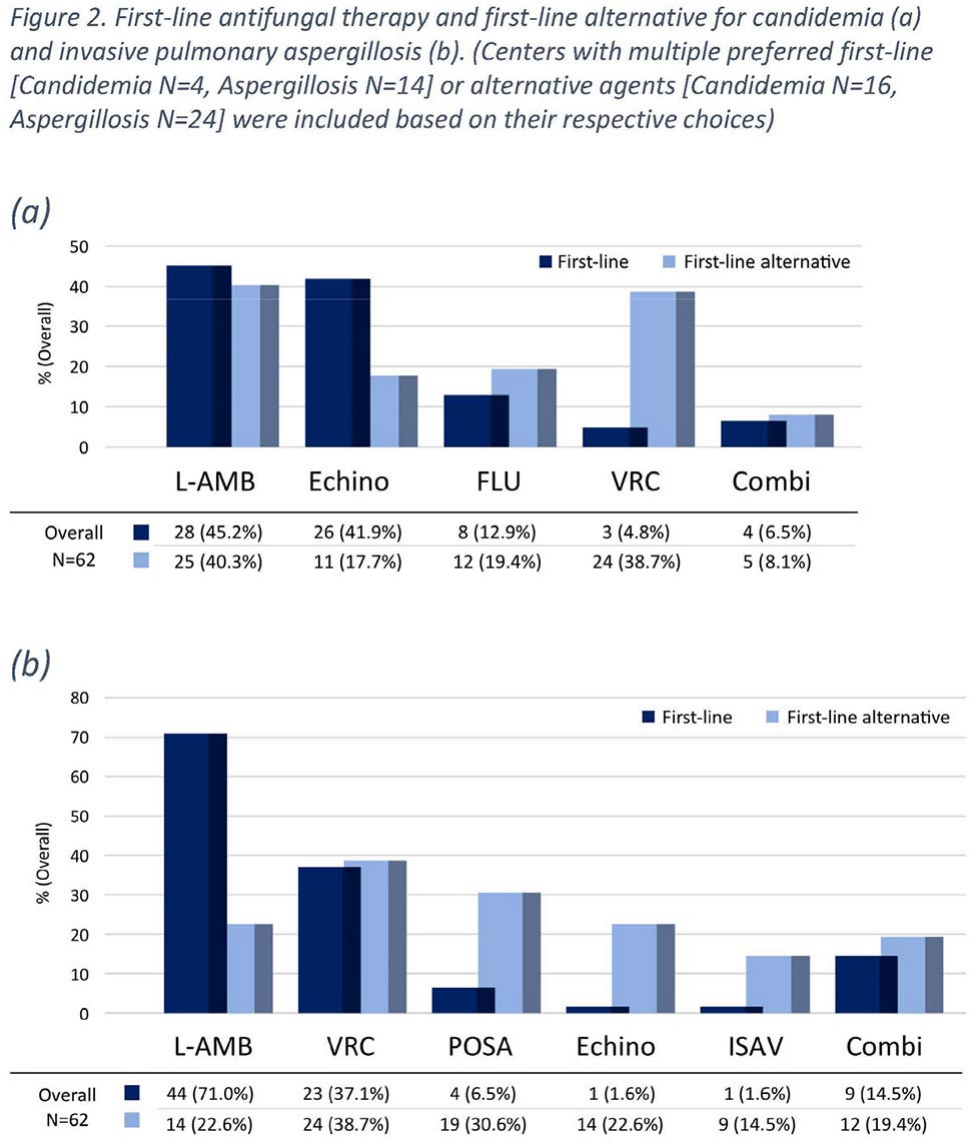

**Methods:**

In Jun-Sep 2024, we surveyed 72 pediatric oncology centers in Germany, Austria, and Switzerland (German Society for Paediatric Oncology and Haematology) using a questionnaire covering center volume, ID expertise, diagnostic tools, prophylaxis protocols, therapeutic drug monitoring (TDM), and treatment pathways for aspergillosis and candidemia. Data were analyzed descriptively; associations between center size, ID resources, and IFD incidence were tested via Mann–Whitney U and linear regression.

**Results:**

Sixty-two centers (86% response) participated: 51 DE, 5 AT, 6 CH (Figure 1). Median new oncology cases in 2023 was 56 (IQR 40–75); 55% managed HCT. Proven or probable IFDs were reported by 89% of centers at a median incidence of 4.6% (IQR 3.0–5.9%). A pediatric ID specialist was available in 58% of centers (100% CH, 51% DE, 40% AT); 58% offered formal ID consultation (24% 24/7). Larger centers more often had ID specialists (p=0.008) and maintained antifungal SOPs (p=0.02). All centers performed culture and histopathology; galactomannan testing in 94%, β-D-glucan in 53%, PCR in 86%. In-house TDM for voriconazole was available in 52%, less frequently for posaconazole and isavuconazole. Prophylaxis strategies varied, with liposomal amphotericin B (AMB) used most frequently across risk groups. AMB was the preferred first-line therapy for invasive pulmonary aspergillosis (71% of centers) and candidemia (45%), followed by voriconazole and echinocandin, retrospectively (Figure 2).

**Conclusion:**

Heterogeneity exists in IFD management across pediatric oncology centers in the DACH region, influenced by center size and ID resource availability. Gaps include inconsistent SOPs, limited 24/7 ID support, and incomplete access to TDM. Strengthening oncology–ID collaborations, standardizing SOPs, and enhancing antifungal stewardship -potentially via digital platforms- may harmonize care and improve outcomes for children at risk of IFD.

**Disclosures:**

Oliver A. Cornely, Prof. Dr., Al-Jazeera Pharmaceuticals/Hikma: Honoraria|Basilea: Advisor/Consultant|Cidara: Advisor/Consultant|Cidara: Board Member|Cidara: Grant/Research Support|Elion: Advisor/Consultant|F2G: Grant/Research Support|Gilead: Advisor/Consultant|Gilead: Grant/Research Support|Gilead: Honoraria|GlaxoSmithKline: Advisor/Consultant|GlaxoSmithKline: Honoraria|Grupo Biotoscana/United Medical/Knight: Honoraria|Melinta: Advisor/Consultant|Melinta: Board Member|MSD: Honoraria|Mundipharma: Advisor/Consultant|Mundipharma: Grant/Research Support|Mundipharma: Honoraria|Pfizer: Advisor/Consultant|Pfizer: Grant/Research Support|Pfizer: Honoraria|Pulmocide: Board Member|Scynexis: Advisor/Consultant|Scynexis: Grant/Research Support|Shionogi: Advisor/Consultant|Shionogi: Honoraria Andreas H. Groll, MD, Basilea: Advisor/Consultant|Gilead: Advisor/Consultant|Gilead: Grant/Research Support|Merck, Sharp & Dohme: Advisor/Consultant|Mundipharma: Advisor/Consultant|Pfizer: Advisor/Consultant|Pfizer: Honoraria

